# Translator’s Preface from the Translation of *the Organon*

**Published:** 1893-01

**Authors:** B. Fincke

**Affiliations:** Brooklyn, N. Y.


### TRANSLATOR'S PREFACE FROM THE TRANSLATION OF *THE ORGANON.*

BY B. FINCKE, M. D.

Motto: "Ich hab's gewagt."—*Ulrich von Hutten.*

The translation here presented to the homœopathicians is a labor of love commenced on New Year's day, 1880. It grew out of the desire to render the diction of Hahnemann in English as closely as possible, to make it understood. Though a difficult and venturesome undertaking, the translation has been made with the fidelity which the piety for the great master of the homœopathic art of healing imposed upon the translator. If the reader stumbles over the long-winded, cumbersome, and involved sentences which meet him at almost every page, and inclines him to throw aside the careful work of many years as "outrageous English," let him beware of his rashness, and acquire the patience which the product of such an eminent mind as that of Hahnemann demands. Let him first study the German language, and after becoming proficient in its expressions in writing, learn that the impatient German scholar likewise might feel justified to throw aside the German original with the similar indignation as the English scholar. But they both would be equally in error, and in following their inclination would do grievous injustice to the master who poured out his homœopathic soul in his immortal work, *The Organon of the Healing Art.*

In the preface to his translation of Hahnemann's writings, Dr. Charles J. Hempel declared: "But for the admirable truths which Hahnemann points out in this volume it would probably never be read in German. Hahnemann's phraseology is so involved and bears so little resemblance to the usual mode of constructing periods, either in German or any other language, that it is utterly impossible to furnish a bare translation of Hahnemann's writings." The same was acknowledged by Dr. Dudgeon, who, in the preface to his translation of *The Organon*, says: "Convinced that what the English student of Homœopathy required was an exact reproduction of the founder's great work, I have conscientiously endeavored to render my translation as literal as possible, and as far as the different genius of the two languages admitted, I have retained the same expressions, figures of speech, and even the somewhat cumbrous and tautological style of the original."

Also, Dr. C. Wesselhœft in the preface to his translation of *The Organon*, coincides with the other translators, when he says: "Each paragraph consists of a single uninterrupted sentence, which, like a ponderous block of stone, hewn and sculptured by the skill of an artisan, seems to have been lifted with Titan power to fill its place and purpose in the structure. It was impossible always to reproduce these sentences in English."

The judgment of these eminent translators indeed testifies to the difficulty of a true translation, but just for that reason the task of rendering the German original into an English text which retains the original structure, style, meaning of the words, and even the words themselves turned into English can and should not be shirked; for here is not the question of "English as she is spoke," but how it is written to give the meaning of Hahnemann's spirit which is conveyed to the mind through the medium of the English language. What Wolfart (*Mesmerismus,* 1814, p. xxxiv) in a similar relation expressed regarding his translation of Mesmer's work from the French, applies with great aptitude here also.

" At any rate," says he, " it will be found on comparing the original text with my translation that I have translated literally as far as it was possible, by which I mean that I even followed the formation and position of the words. A true translation must just as well render the sense as the color and shape of the expression; in short, the style, because certainly the original sense cannot be rendered without the original mode of expression, and because here it was of especial importance to retain the unpretending and unadorned mode of demonstration."

To this may be added another testimony from Roget's *Thesaurus of English Words and Phrases* (new edition. De Wolfe, Fiske & Co., Boston. Introduction, p. xv):

" The utility of the present work will be appreciated more especially by those who are engaged in the arduous process of translating into English a work written in another language. Simple as the operation may appear, on a superficial view, of rendering into English each of its sentences the task of transfusing with perfect exactness the sense of the original, preserving at the same time the style and character of its composition, and reflecting with fidelity the mind and the spirit of the author is a task of extreme difficulty."

If, then, the justification of translating the style of the original as close as possible is admitted, the homœopathician entering upon its intricacies will be rewarded by entering at the same time upon the spirit in which Hahnemann wrote, and he will understand him better than by studying the translations which, with Dr. Dudgeon allowed a literal rendering only "as far as the different genius of the two languages admitted, " or with Dr. C. Wessehœft found " that plain English expressions and simplicity of style were needed to render the work accessible to the student " on account of which he avoided " too close an adherence to Hahnemann's construction, style, and punctuation," or with Dr. Hempel rendered " not words, but ' ideas ' in first mastering the sense of the period and afterward embodying it in the foreign tongue in a free manner."

These translators, as it were, let the Hahnemannian light shine through their own mental medium, a natural process by which that light in its path must suffer more or less refraction. This in the present translation has been avoided, to the evident disadvantage of the translator, whose aim was to make the original German as English as it was possible to do. If the student, unable to get at the fountain-head in its originality wants to imbibe the very spirit of it, the very nearest must be a literal translation such as here has been attempted with studious care. Such a study will be an "open sesame" to him, leading to original views such as are not yet taught in the colleges, but which every one for himself must dig out of the original text. Even if the homœopathician understands the German text well it will tend to let him see many things in a different and new light when he looks at them clothed in his own native language. Moreover, why should the student if he complains of the un-English rendering of Hahnemann not take the pains to construe his sentences as he does in his study of the old Greek and Latin classics? The reading of *The Organon* deserves these pains just as well.

In this connection the existing translations, every one of which has a merit of its own, may, with advantage, be used as collaterals of the present translation for the purpose of a better understanding of the original text.

Such considerations must also excuse many apparent crudities which, if fidelity was required, could not be changed into perhaps more elegant or more correct expressions. The question always was: Did Hahnemann actually write this? If he did, it is none of the translator's business to change it, and even if it does not seem to the writer's credit, the translator must abide by it for the sake of truth. Hahnemann has expressed his thought in this manner. If the translator thinks he could have expressed it in another manner and perhaps better, yet he is not entitled to make the change, for he must presuppose that Hahnemann himself would have done so if he wanted to. The great admiration and piety which with all homœopathicians the translator bears for Hahnemann can nevertheless give him no right to change a tittle of what he wrote. Where the text could not be rendered with elegance, this has been sacrified to correctness.

As to the intrinsic merit of *The Organon* it is not necessary to say anything to enhance it. Since societies have been founded in this country which set themselves to work of studying *The Organon,* the value of this unique text-book of the homœopathic art and science has been more and more recognized, and it has been laid down as an axiom that no student of homœopathics can accomplish anything creditable in the way of healing if he does not go up to the fountain-head, to the earnest study of Hahnemann's *Organon of the Healing Art.*

BROOKLYN, N. Y., on Independence Day, 1892.

